# Knowledge, Attitude, Practice, and Determinants Emergency Contraceptive Use among Women Seeking Abortion Services in Dire Dawa, Ethiopia

**DOI:** 10.1371/journal.pone.0110008

**Published:** 2014-10-17

**Authors:** Meskerem Abate, Nega Assefa, Tadesse Alemayehu

**Affiliations:** 1 Sabian Health Centre, Dire Dawa Health Office, Dire Dawa, Ethiopia; 2 College of Health and Medical Sciences, Haramaya University, East Harerge, Ethiopia; Karolinska Institutet, Sweden

## Abstract

**Background:**

Unplanned pregnancy from casual sex, unplanned sexual activity, and sexual violence are increasing. Emergency Contraceptives (EC) are used to prevent unplanned pregnancies thereby preventing the occurrence and consequences of unplanned pregnancy. Emergency contraception is widely available in Ethiopia particularly in major cities. Yet the use of EC is very low and abortion rate in cities is high compared to the national average.

**Objectives:**

To assess knowledge, attitude and practice and determinants on the use of emergency contraception among women obtaining abortion service at selected health institutions in Dire Dawa, Eastern Ethiopia.

**Methods:**

A facility based cross-sectional study was conducted on 390 women selected by multi-stage random sampling technique. The samples were generated from government and private for non profit health facilities. Participant’s knowledge and attitude towards emergency contraception were measured using composite index based on 7 and 9 questions, respectively and analyzed using mean score to classify them as knowledgeable or not, and have positive attitude or not. Practice was assessed if the women reported ever use of emergency contraception. Determinants of use of emergency contraception were analyzed using logistic regression.

**Result:**

Out of 390 women interviewed, 162 women (41.5%) heard about EC, only 133 (34.1%) had good knowledge, and 200 (51.3%) of the respondents had positive attitudes towards to EC. Ever use of EC was reported by 38 (9.7%). Age, living arrangement, education, marital status, religion were found to be significantly associated with the use of emergency contraceptives. Women with poor knowledge were less likely to use EC compared to the knowledgeable ones [AOR = 0.027, 95% CI (0.007, 0.105)].

**Conclusion:**

The study identified that most respondents lack adequate knowledge on the method of EC. In addition ever use of EC is very low.

**Recommendations:**

Health professions should give attention in increasing knowledge and uptake of Emergency Contraception.

## Introduction

Emergency contraceptives (EC) is the only method women can use to prevent pregnancy after they have had unprotected sexual intercourse, have experienced a contraceptive failure, have remembered too late that they have forgotten to take their birth control pills, or have been forced to have sex against their will. EC is sometimes referred to as “morning-after” or “post-coital” contraception. EC is intended for occasional or emergency use only and not as a regular means of contraception. Formerly, EC was thought to be effective only within 72 hours, but recent studies have confirmed it is effective for up to 120 hours [1].The copper-releasing intrauterine device (IUD) can be used safely for EC up to 5 days after unprotected intercourse, reducing the risk of pregnancy by over 99 percent [2].

EC is largely underutilized worldwide and has been referred to as one of the best kept secrets in Reproductive Health [3]. Globally, use of EC is relatively low. In the United States usage has been reported as 9.4%, in South Africa as 4% and in Iran as 5.2% [4], [5], [6]. In addition, studies have showed that knowledge, and attitude on emergency contraception among women are limited [7].

Each year globally about 250 million pregnancies occur. One-third of these pregnancies are unintended, of which 20% terminated by induced abortion. In low income countries, more than one-third of the 182 million pregnancies are unintended; of which 19% are terminated by induced abortion. Among the induced abortion 11% are unsafe [8].

Due to high unmet need for family planning and its subsequent effect, many women in Ethiopia are experiencing the challenges of abortion and unwanted child birth. As a result, the Federal Ministry of Health of Ethiopia has allowed the distribution of EC in drug stores and the provision of safe abortion services in medical setup for those who demand the service under certain conditions such as rape, incest, sexual violence, etc.

In Dire Dawa, EC pills can be found in any drug retails in a two dose oral pills that should be taken in 12 hours apart. According to the information on the leaflet distributed with the drug, the two doses should be taken within 72 hours after exposure to unprotected sex. In major cities, in Ethiopia, even though EC is easily accessible from drug stores, abortion rate is quite high. For example, the abortion rate in Dire Dawa, is 184 per 1,000 per women of reproductive age, while the national average is 49 per 1,000 [10]. For this, many explain that Dire Dawa is a city, and there are plenty of health facilities, both public and private that render abortion service. Therefore, it is reasonable to have this difference between national average and the city’s value.

Abortion, even if performed in safe way, it may be painful, and may have psychological and physical stress. Here, the paradox is while EC service is widely available, why would women prefer to have an abortion in the face of potential problems associated with the procedure. For this, several reasons can be given, yet the dominant one is lack of adequate knowledge on EC among the general public and women in particular.

This study assessed knowledge, attitude and practice (KAP) and determinants of use of emergency contraceptive among women obtaining abortion service in Dire Dawa at selected health institutions. The outcome of this study will be used to put interventions in place in order to increase the use of EC thereby reverse the occurrence of unwanted pregnancy and its sequelae.

## Methods and Materials

Facility based cross-sectional study was conducted from January to February 2014 among women who obtained abortion service in selected health facilities in Dire Dawa city. Dire Dawa city is located in the Eastern part of Ethiopia 505 kms from Addis Ababa, the capital of Ethiopia. Based on the 2007 census conducted by the central statistical agency of Ethiopia, Dire Dawa Administrative council has a total population of 341,834 of whom 171,461 are males and 170,373 are females and the growth rate was 2.5% [11].

The sample size was calculated assuming proportion of EC knowledge of married and unmarried women experienced induced abortion to be 9% and 17.5%, respectively, obtained from previous study in Jimma, Ethiopia [12]. A 5% margin of error, 95% confidence level and adding a non-response rate of 10%. Accordingly, a sample size of 431 was obtained.

A multi-stage sampling technique was used to select 431 study participants. A list of health facilities prepared and the facilities were stratified into government and private for non-profit health facilities. Then they were selected by simple random sampling from the list. The sample size to each selected facility was allocated proportionally using client volume in the facilities served in the year 2013. All women in reproductive age group who came for obtaining abortion service in the selected health facilities during the study period were source of population for the study.

A pre-tested structured questionnaire was used to collect data. The questionnaire was translated into three local languages (Amharic, Oromifa and Somali) and then back to the original English to maintain its consistency. The questionnaire includes socio-demographic variables, knowledge, attitude and practice questions about EC. Seven female diploma nurses collected the data by face-to-face interview. The data collection was supervised by Nurses who have bachelor degree.

Following data collection a unique code was assigned to each questionnaire and checked for completeness then entered into computer on EPI-info software version 3.5.1. Data was cleaned and analyze using Statistical Package for Social Science (SPSS) version 16.1. First, descriptive analysis was done to identify the level knowledge, attitude and practice which is expressed in terms of frequencies and percentages. Knowledge of EC was assessed using questions of awareness about the types, mentioning the time limit for EC use after unprotected sex and responding the dosage of emergency contraceptive. Attitude was assessed using the responses of the way clients were thinking or behaving about EC. Practice was determined based on ever use of EC after exposure to unprotected sexual intercourse to prevent unintended pregnancy.

Seven questions were used to measure knowledge of the respondents on EC. If respondents get the right answer, it was coded as Yes “1” if not it was coded as No “0”. The respondent’s knowledge scores were aggregated and ranged 0–7. Based on the cumulated score, respondents, who scored above the mean value, were considered as “knowledgeable”; while those who score below the mean were considered as “not knowledgeable”.

The respondents’ attitude was measured using nine items rated on a five-point Likert scale that was later changed to dichotomous outcome. The scores were aggregated and ranged 0–9. Based on the cumulative score, respondents who scored above the mean of the total were considered as having “positive attitude”; while those scored below the mean of the total were considered as having “negative attitude”. Finally, odds ratios with 95% CI were calculated to measure risk and statistical significance of socio-demographic, knowledge and attitude towards use of EC. Logistic regression analysis was used to assess the relative effect of independent variable on the dependent variables.

The study was carried out after obtaining ethical clearance from Institutional Ethical Clearance Board (IRB) of College of Health Sciences, Haramaya University. All participants were informed about the objectives and their right whether to choose to participate or not and their right to leave the study at anytime they wish. A written informed consent was obtained from all participants. Study participants, whose age is less than 18 years, assent was taken from the study participants. For this study group, as the issue is very sensitive, consent was not obtained from the care taker/guardian. All data, consent/assent documents are kept in safe place at the office of the first author. This procedure of taking consent and assent was accepted by the IRB, as the survey took only oral responses. In order to keep confidentiality of respondents’ information, only data collector and supervisor were involved in the data collection and supervision process.

## Result

### Socio – Demographic Characteristics of the Respondents

Among women who came for abortions service at the selected health facilities, a total of 390 women were interviewed. The response rate was 90.1%. The minimum and maximum age of the respondents was 16 and 41 respectively. Among the respondents 278 (71.3%) were in the age range of 20–29 years. Regarding educational status, 38.2% had some secondary level education, 32.1% to tertiary level, 24.3% to primary level and 5.4% had no education (Table 1).

**Table 1:** Socio-demographic characteristics of women who came for induced abortion in Dire Dawa town, March 2014.

Variables	Frequency	Percent
**Age**		
15–19	72	18.5
20–24	186	47.7
25–29	92	23.6
30+	40	10.3
Total	390	100
**Living arrangement**		
Parent	148	37.9
Spouse	139	35.6
Friend	42	10.8
Alone	61	15.6
Total	390	100
**Occupation**		
Student	159	40.8
Merchant	32	8.2
House Wife	70	17.9
Commercial Sex Worker	16	4.1
Employee	113	29
Total	390	100
**Education**		
Illiterate	21	5.4
Primary Education	95	24.3
Secondary Education	149	38.2
Diploma and above	125	32.1
Total	390	100
**Marital Status**		
Unmarried	251	64.4
Married	139	35.6
Total	390	100
**Religion**		
Christian	220	69.2
Muslim	120	30.8
Protestant	53	13.6
Catholic	6	1.5
Total	390	100

### Knowledge on EC among Women who Seek Induced Abortion

Knowledge scores were aggregated and ranged 0–7 with mean 2.04, median 1 and SD 2.96. Based on this result, the summary index for knowledge towards EC showed that 133 (34.1%) women were knowledgeable while 257 (65.9%) were not knowledgeable. When asked about the options available to prevent unintended pregnancy, 249 (63.8%) responded abortion while 114 (29%) reported the use of EC. Regarding EC methods majority 142 (36.4%) respondents mentioned ECP while only one (0.01%) mentioned IUCD. Concerning the appropriate time for taking EC and doses, 72 of them described within 72 hours; 66 women stated two times are the appropriate doses of ECP. Similarly, 74 respondents mentioned 12 hours is the time intervals to take ECP. Regarding the main sources of information about EC, friends and health workers were the main sources (Table 2).

**Table 2:** Knowledge of Emergency Contraceptive among women attending abortion service in Dire Dawa town, March 2014.

Variables	Frequency	Percent
**Things to do after unprotected** **sexual intercourse (correctly replied)**	114	29.2
*Use ECP*	114	29.2
Abortion	249	63.8
No alternative	6	1.5
I don’t know	21	5.4
Total	390	100
**Types of EC methods(correctly replied)**	142	36.4
*ECP*	142	36.4
*IUCD*	1	0.3
COC	0	0
Depo	19	4.9
I don’t know	228	58.5
Total	390	100
**Time limit to take EC(correctly replied)**	72	18.5
Any time	8	2.1
Before sex	44	11.3
24 hours after sex	28	7.2
*72 hours after sex*	72	18.5
120 hours after sex	5	1.3
I don’t know	233	59.7
Total	390	100
**Dose of ECP (correctly replied)**	66	14.6
One dose	57	14.6
*Two dose*	*66*	*16.9*
Three dose	9	2.3
I don’t know	258	66.2
Total	390	100
**Time interval between doses (correctly replied)**	74	19.0
*12 hours*	*74*	*19.0*
24 hours	31	7.9
I don’t know	285	73.1
Total	390	100
**Advantage of EC (correctly replied)**	*106*	*27.2*
*Prevent pregnancy*	*106*	*27.2*
Regular contraceptive	5	1.3
Regulation of menstrual cycle	54	13.8
Abortion	25	6.4
I don’t know	200	51.3
Total	390	100
**Situations of requirement EC to prevent unintended pregnancy(correctly replied)**	101	25.9
*Raped*	13	3.3
*Condom breaks*	27	6.9
*Regular pills are missed*	21	5.4
*Use no contraceptives*	66	16.9
***In all above situations***	***101***	***25.9***
I don’t know	162	41.6
Total	390	*
**Knowledge of Emergency Contraceptive (summary index)**		
**Not Knowledgeable**	257	65.9
**Knowledgeable**	133	34.1
Total	390	100%

*Percentage could be greater than 100 since multiple responses are possible.

NB*: Italicized fonts are correct responses.*

### Attitudes of women attending abortion service towards EC

Attitude scores were aggregated and ranged 0–9 with mean 6.13, median 7 and SD 2.42, Based on this, the overall summary index for attitude assessment towards emergency contraceptive revealed that 51.3% (200) of the respondents had positive attitude towards use of EC (Table 3).

**Table 3:** Attitude towards Emergency Contraception among abortion service users in Dire Dawa town, March 2014.

Variables	S. disagree	Disagree	Neutral	Agree	S. Agree	Total
	%	%	%	%	%	%
I would use ECP if Ihave unprotected sex	0.0	7.7	10.8	80.0	1.5	100
EC methods are safe	1.0	20.0	17.4	60.5	1.0	100
I would recommendEC methods to a friend	1.3	30.0	11.8	53.1	3.8	100
EC methods should be limitedbecause they could have side effect	5.4	62.3	11.0	18.7	2.6	100
Increased accessibility of ECbrings about irresponsible sexual behavior?	9.2	56.4	15.4	17.0	1.0	100
My partner has positive attitudetowards EC methods	0.3	23.8	12.6	62.6	0.8	100
If male partner knows about EC,he may be less likely to use condoms	0.8	67.9	6.7	24.1	0.5	100
Increased accessibility of ECmake women stop using other forms of contraceptive	9.2	61.5	9.7	18.5	1.0	100
EC methods could have aneffect on future fertility	7.2	68.5	13.1	10.8	0.5	100
**Attitude towards EC (Summary index)**	**Number**	**%**				
**Negative attitude**	190	48.7				
**Positive attitude**	200	51.3				

### Emergency Contraceptive Use

Out of 390 respondents, 38 (9.7%) mentioned that they had ever used ECP at least once. Bad rumour (58), fear of side effect (41), religious prohibition (16) and husband influence (7) were the reasons mentioned by the respondents for not using EC while 228 women never heard of EC and two respondents said that they do not know where EC is available. Among the 38 women who ever used ECP, 24 (63.2%) of them were between the age group of 25–29.

Regarding the living arrangement of those, who ever used EC, 19 live with their parents, 10 live with spouse, three live with friend and six of the respondent live alone. Twenty four of the women were employee while nine of them were students and the remaining six were merchants, housewives, or commercial sex workers. Regarding educational background of the women who ever used ECP, 33 had diploma & above while three had some secondary education and two had some primary education. Of the 38 respondents who ever used EC, 28 women were unmarried; and 35 women were Christian.

### Determinants on the use of Emergency Contraceptives

Socio demographic factors, knowledge and attitude found to be significantly associated on the use of EC. Accordingly, age of respondents was found significantly associated with the use of EC [AOR = 0.2, 95% CI (0.01, 0.8)]. The likelihood of EC usage decreased as the age of the women increased. Women of the age group 20–24 were less likely to use EC compared with the younger age group (15–19) with an AOR 0.18 [CI 0.04, 0.8]. Similarly women who were living with their spouse were less likely to use EC compared to those living alone, AOR 0.001 [0.000, 0.017].

Religion of the respondents was found to be significantly associated with the use EC [p = 0.024]. The study shows that more Christian respondents were using EC than that of Muslim 4.113 [1.205, 14.039]. Marital status significantly associated with the use of EC [p = 0.000]. Married women were less likely to use EC compared to unmarried women. Education is significantly associated with use of EC. The likelihood of using EC increased as the education level of the study subjects increased. Those respondents with diploma & above were more likely to use EC compared to primary and secondary education. Knowledge of respondents towards EC was significantly associated with use of EC [p = 0.000]. The result showed that the likelihood to use EC increased among the knowledgeable respondents. Respondents who had poor knowledge of EC were found less likely to use EC. In the crude analysis, attitude is significant to towards EC [COR = 0.1, 95% CI (0.1, 0.14)]. But it is insignificant after adjusting for possible confounders [p = 0.291] (Table 4).

**Table 4:** Determinants of the use Emergency Contraception in Dire Dawa town, March 2014.

Variables	Practice				
	Yes	No	COR (95% CI)	AOR (95% CI)	P-Value
**Age**					
15–19	3 (0.8)	69 (17.7)	1	1	0.001
20–24	6 (1.5)	180 (46.2)	**0.226 (0.061, 0.833)**	**0.18 (0.040, 0.821)**	0.027
25–29	24 (6.2)	68 (17.4)	1.727 (0.451, 6.621)	1.667 (0.331, 8.386)	0.535
30+	5 (1.3)	35 (9.0)	1.607 (0.319, 8.092)	1.935 (0.256, 14.642)	0.522
**Living arrangement**					
Alone	6 (1.5)	55 (14.1)	1	1	0.000
Parent	19 (4.9)	129 (33.1)	3.251 (0.974, 10.857)	1.974 (0.464, 8.217)	0.350
Spouse	10 (2.6)	129 (33.1)	**0.002 (0.000, 0.021)**	**0.001 (0.000, 0.017)**	0.000
Friend	3 (0.8)	39 (10.0)	3.509 (0.682, 18.060)	3.371 (0.494, 23.027)	0.215
**Occupation**					
Employee	24 (6.2)	89 (22.8)	1	1	0.005
Student	9 (2.3)	150 (38.5)	0.334 (0.109, 1.021)	0.427 (0.112, 1.634)	0.214
Merchant	2 (0.5)	30 (7.7)	3.304 (0.786, 13.893)	5.014 (0.881, 28.552)	0.069
House wife	2 (0.5)	68 (17.4)	0.474 (0.124, 1.817)	0.365 (0.062, 2.147)	0.265
Commercial sex worker	1 (0.3)	15 (3.8)		*NI	
**Education**					
Diploma & above	33 (8.5)	92 (23.6)	1	1	0.000
Illiterate	0	21 (5.4)		**NI	
Primary Education	2 (0.5)	93 (23.8)	**0.056 (0.013, 0.241)**	**0.055 (0.010, 0.293)**	0.001
Secondary Education	3 (0.8)	146 (37.4)	**0.059 (0.017, 0.305)**	**0.059 (0.014, 0.248)**	0.000
**Marital Status**					
Unmarried	28 (7.2)	223 (57.2)	1	1	
Married	10 (2.6)	129 (33.1)	**0.002 (0.000, 0.022)**	**0.002 (0.000, 0.020)**	0.000
**Religion**					
Christian	35 (9)	235(60.3)	1	1	
Muslim	3(0.8)	117 (30)	1.853 (0.711, 4.832)	**4.113 (1.205, 14.039)**	0.024
**Knowledge**					
Knowledgeable	35 (9)	98 (25.1)	1	1	
Not Knowledgeable	3 (0.8)	254 (65.1	0.116 (0.073, 0.183)	**0.027 (0.007, 0.105)**	0.000
**Attitude**					
Positive attitude	34 (8.7)	166 (42.6)	1	1	
Negative attitude	4 (1)	186 (47.7)	0.080 (0.046, 0.137)	0.605 (0.238, 1.537)	0.291

*NI = Not included as the observations are below 20.

**NI = Not included as the observation group does not use EC.

## Discussion

In this study 34.1% of study participants were knowledgeable on Emergency Contraception. The study also revealed that 51.3% of the respondents had positive attitudes towards EC. Ever use of EC was 38 (9.7%). Knowledge is significantly associated with the use of EC [AOR = 0.027, CI 95% (0.007, 0.105)]. Attitude towards EC found to be insignificant [AOR = 0.605, CI95% (0.238, 1.537)].

Strength of this study is that the respondent’s willingness to participate in the study and obtaining the response rate of 90.1%. Some of the limitations are, it is focused on women who were seeking abortion, a specific population with failure in preventing pregnancy; it may not reflect the general condition of women in the city. In addition, the study populations from private health facilities were not included in the study so that this study might not be representing the entire population.

Less than half of the women had heard about EC among which, majority mentioned Emergency Contraception Pill as EC method, while only one woman mentioned Intra Uterine Contraceptive Device. Only 72 women of the respondents correctly identified 72 hours as the time limit for the method use. The study also identified that knowledge of respondents towards EC was significantly associated with the use of EC. Those respondents with poor knowledgeable were less likely to use EC compared to those with good knowledge. The study also indicated that knowledge of unmarried (72.2%) women towards EC was higher than that of married (27.8%). This finding is similar to the study conducted in Jimma (17.5% unmarried and (9%) married [12].

Nearly half of the study participants had a positive attitude towards emergency contraceptives. Although educated women generally have a positive attitudes towards EC, considerable number of women had inappropriate information about EC. Of which only 62 (62%) respondents had appropriate knowledge about EC while 38 (38%) respondents lack adequate information. In a similar study conducted in Kampala, Uganda, 29% of the respondents had inappropriate information [13]. The use of EC among those respondent with negative attitude compared to positive attitude was insignificant [p = 0.291]. The reason could be lack of information on the use and options of EC despite respondents’ positive attitude to use of EC. This study also showed that, health care providers were not providing information about the availability and methods of EC to their clients. Out of the 390 respondents, only 34 (8.7%) women mentioned that they were informed by health care providers about EC. This result is almost similar to the study conducted in Nigeria which was 10% [14].

Less than ten percent of the study participants mentioned ever use of EC. This result is slightly higher than the result obtained from Jimma 5.4% [12]. The reason could be associated with the education of the women as most respondents (86%) in this study who ever used ECP had diploma and above. Of the 38 respondents who ever used ECP, majority (35) were Christians while three were Muslim.

Married women were less likely to use EC compared to unmarried women. This finding is different from the study conducted among the college students in Arba Minch town [15]. The result of this study showed that education is significantly associated with the use of EC. The likelihood of EC use increased as the level of education of the study participants increased. Those respondents with diploma & above were more likely to use EC compared to both primary and secondary education level.

Source of information was highly associated to the use EC. In the study, the major source of information was friends. This result is consistent to the study finding in Lusaka, Zambia where majority referred friends as a major source of information for EC. The same study revealed that women who obtained information from friends were less likely to use EC compared to those women who heard from health workers [16].

## Conclusion and Recommendation

In conclusion the great potential of emergency contraceptives to prevent unintended pregnancies and induced abortions was not considered as an option by the study participants. The use of EC is very low. The major reason for this is lack of adequate knowledge on the method and its availability. IUCD as EC is not known at all. This study showed that information on EC is not provided adequately by the health professionals. Hence, there is a need to educate the community, in particular women of reproductive age about ECs. Education on methods available, the correct time limit for use, and accurate message about its effect on health through health professionals and mass media should be given. The health executives should give due attention to design strategies and strengthening the health education in all health facilities as well as high schools and colleges focusing on the availability and options of Emergency contraceptives.
